# The Clonal Trajectory of Liver and Lung Metastases in Pancreatic Ductal Adenocarcinoma

**DOI:** 10.1002/cnr2.70228

**Published:** 2025-05-09

**Authors:** Ofer N. Gofrit, Ben Gofrit, Aron Popovtzer, Jacob Sosna, S. Nahum Goldberg

**Affiliations:** ^1^ Department of Urology Hadassah Medical Center, Faculty of Medicine, Hebrew University of Jerusalem Jerusalem Israel; ^2^ School of Engineering and Computer Science, Hebrew University of Jerusalem Jerusalem Israel; ^3^ Department of Oncology Hadassah Medical Center, Faculty of Medicine, Hebrew University of Jerusalem Jerusalem Israel; ^4^ Department of Radiology Hadassah Medical Center, Faculty of Medicine, Hebrew University of Jerusalem Jerusalem Israel

**Keywords:** clonal origin, liver metastases, lung metastases, pancreatic ductal adenocarcinoma

## Abstract

**Background:**

Metastatic spread can follow either the linear route‐dissemination of fully malignant cells from the primary tumor, or the parallel route‐dissemination of immature tumor cells and independent maturation to metastases in target organs. The linear/parallel ratio (LPR) is a model that uses metastases diameter comparisons to decipher dissemination route. LPR of +1 suggests pure linear and −1 pure parallel spread.

**Aims:**

To examine the metastases trajectory in pancreatic duct adenocarcinoma (PDAC).

**Methods and Results:**

A total of 133 patients with PDAC, including 97 patients (72.9%) with synchronous and 36 (27.1%) with metachronous metastases with a total of 1054 lung and 2898 liver metastases, were evaluated. We found that metastatic spread to both liver and lungs is almost exclusively via the linear route (lungs median LPR + 1, interquartile range [IQR] 0.97,1. Liver median LPR + 0.98, IQR 0.83,1). Calculated from the primary diagnosis, overall survival (OS) of patients with metachronous metastases was significantly better compared to patients with synchronous disease (14 months, IQR 10,26, vs. 7 months, IQR 6,9, *p* < 0.0001). However, calculated from the time of metastases diagnosis, OS of both groups was similar (4 months, IQR 3,8, vs. 7 months, IQR 6,9, *p* = 0.235).

**Conclusion:**

These two observations suggest that metastatic spread of PDAC is almost exclusively via the linear route, that is, directly from the primary tumor. Therefore, liver or lung metastases are already present in most patients with PDAC at the time of initial diagnosis. This suggests that local treatment in patients with seemingly localized disease does not decrease their risk of developing metastases and that systemic treatment must follow.

## 1 Introduction

Pancreatic ductal adenocarcinoma (PDAC) is the fourth leading cause of death from cancer in both men and women. Its incidence is rising, and it is expected that by 2030 it will become the second cause of cancer death [1]. At presentation, 80% of the patients suffer from advanced disease, and only 4% will live 5 years after the diagnosis [2]. Even after surgery with curative intent, the recurrence rate is high, resulting in a 5‐year overall survival (OS) of only 18%–27% [3, 4, 5]. The liver, peritoneum, lungs, pleura, bones, and adrenal gland are the most common sites of pancreatic cancer metastases [6]. The liver, lungs, and peritoneum are the most common sites of recurrence after surgery with curative intent [7, 8, 9].

The management of advanced PDAC usually does not include surgical resection, as is often the case in other cancer types (e.g., colorectal cancer). Thus, the availability of tissue for research in PDAC is limited [6, 10, 11]. The metastatic process is extremely complex, but for simplicity's sake, metastatic spread is usually considered as following linear or parallel trajectories [12, 13, 14, 15]. The linear pathway assumes accumulation of genetic and epigenetic alterations within the primary tumor. Once a clone has obtained all the necessary properties needed to become a metastasis (invasion, angiogenesis, etc.), it disseminates as a wave of cells that resemble each other and are close genetically to the primary tumor. Additional waves of metastases may follow the first one. Since linear metastases dispatch at the same time and are genetically identical, when landing in a similar soil (e.g., the lungs) they are expected to grow at a similar rate and to reach an almost identical diameter at any time point. Therefore, in linear spread, a single or several clusters of diameters are expected in target organs, according to the number of dispatched waves of metastases. On the other hand, the parallel model assumes early spread of less advanced disseminated tumor cells (DTCs) and independent maturation of these cells into metastases in the target organs. Since DTCs develop independently, they are expected to be genetically diverse and thus, it is expected that they would grow at different rates and reach varied sizes at any time point. Therefore, it would be impossible to cluster parallel metastases according to their diameters.

The linear/parallel ratio (LPR) is an attempt to quantify metastases trajectories. It is the ratio of metastases that can be clustered into groups with identical diameter to the total number of metastases. The number of clusters equals the number of metastatic waves. An LPR ratio of +1 means that all metastases can be clustered and suggests pure linear progression, while an LPR of −1 indicates that none of the metastases can be clustered and suggests pure parallel progression. This concept allows rapid classification of multiple metastases in many patients. In a study of lung metastases, it was found that the trajectory is mostly linear in malignancies of the pancreas, prostate, and thyroid (median LPR of +1). Tumors of the kidney, melanomas, colorectum, and breast show lower LPRs (median of 0.91–0.58), and tumors of the bladder and sarcomas have the lowest LPR (median of 0.5–0.43) [16]. In this study, using the LPR concept we attempted to depict the trajectory of PDAC lung and liver metastases in different clinical situations. This information can potentially shed light on the timing of metastases and direct treatment program.

## 2 Methods

### 2.1 Patients

The files of patients with PDAC (ICD‐9‐CM code 157.0) and either lung metastases (ICD‐9‐CM code 197.0) or liver metastases (ICD‐9‐CM code 197.7), diagnosed between the years 2000 and 2020, were evaluated retrospectively. Data regarding presentation, treatment, and course was obtained. Patient's computerized axial images of the chest and abdomen (preferably with intravenous contrast enhancement) were reviewed, and the largest diameters of each liver and lung metastases was manually measured. The complete database is available in Data S1. The study was approved by the local IRB (approval number 0034‐23‐HMO). Patient's records were anonymized prior to analysis, and no informed consent or additional ethical committee review was required.

### 2.2 Data Analysis and Calculation of the LPR

Metastases were clustered to waves according to their diameters. A lung metastases wave was defined as all metastases with a diameter difference of 1 mm or less, and a liver metastases wave as all metastases with differences of 3 mm or less (due to the lower precision of liver measurement) were clustered into groups. The LPR was calculated by the formula LPR = (*∑*c − *∑*i)/(*∑*c + *∑*i). *∑*c is the sum of metastases that can be clustered according to their diameter, and *∑*i is the sum of isolated metastases (that cannot be clustered). LPR was calculated with a computer code using Python programming language version 3.10 for Microsoft Windows (the code is available in Supporting Information S2). Other parameters examined were the total number of metastases in each patient, the average size of metastases, the size of the largest metastases, the number of metastatic clusters in each patient, and the number of isolated metastasis (that cannot be clustered). Synchronous metastases were defined as metastases diagnosed at the same time as the primary site, and metachronous metastases at any time later. OS rate was calculated twice: once from the time of primary diagnosis and once from the time of metastases diagnosis. Survival rates were calculated from Kaplan–Meier curves and compared using the Wilcoxon test. Continuous variables were compared with a two‐tailed student's t‐test. A *p* value < 0.05 was considered significant.

## 3 Results

Data regarding the course of 133 patients with metastatic PDAC treated over the study period was analyzed. Table 1 shows patients' basic features. Synchronous metastases were found in 97 patients (72.9%) and metachronous in 36 patients (27.1%). After a median follow‐up of 8 months (interquartile range (IQR) 3,16), 120 patients (90.2%) died. In 118 patients (98.3%) death was disease specific, so overall and disease‐specific survivals are almost equal. Median OS (OS) rate was 9 months (96% CIs 7, 10). OS rates after 6, 12, 24, and 36 months were 64.2%, 33.0%, 16.4%, and 7.3%, respectively.

**TABLE 1:** Basic features of the patients.

Mean age (SD)	66.2 years (10.3)
Female/male	53 (39.8%)/80 (60.2%)
Location of primary cancer	
Head	92 (69.2%)
Body	18 (13.5%)
Tail	23 (17.3%)
Timing of metastases	
Synchronous	97 (72.9%)
Metachronous	36 (27.1%)
Metastasis's locations^a^	
Liver only	80 (60.1%)
Lungs only	27 (20.3%)
Both liver and lungs	26 (19.6%)

^a^ Considering only the liver and lungs.

### 3.1 Prognostic Factors

**FIGURE 1:**
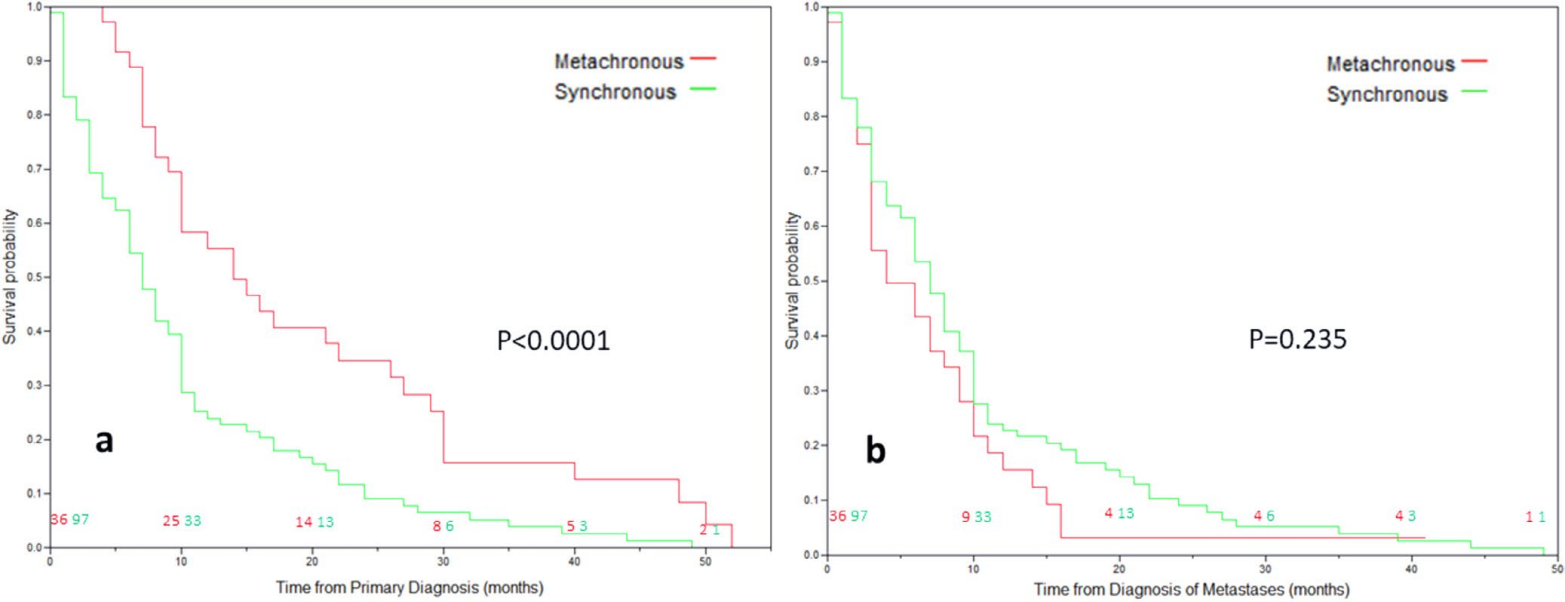
Overall survival curves of patients with synchronous and metachronous metastases. Measured from the time of primary diagnosis (a) and from the time of metastases diagnosis (b).

Table 2 and Figure 1 present the effect of various prognostic factors on OS rate. Patient's gender and location of the primary tumor had no significant effect on OS rate. Metachronous metastases developed after a median period of 7.5 months (IQR 4.25,15.75). When calculated from the time of primary diagnosis, the OS rate of patients with metachronous metastases was significantly better compared to patients with synchronous disease (14 months, IQR 10,26, vs. 7 months, IQR 6,9, *p* < 0.0001). However, when calculated from the time of metastases diagnosis, the OS rate in both groups was similar (4 months, IQR 3,8, vs. 7 months, IQR 6,9, respectively, *p* = 0.235). Prognosis according to metastatic site is presented in Table 2 and Supporting Information S3. The OS of patients with liver‐only disease was significantly worse compared to patients with lung‐only metastases (median OS rate of 8 months, IQR 6,10 vs. 10 months, IQR 7,22, *p* = 0.032), but the survival curve of patients with both liver and lung metastases was almost identical to that of patients with lung‐only disease (median of 10 months, IQR 7,22).

**TABLE 2:** The effect of various prognostic factors on overall survival rate.

	Median overall survival (95% CIs)	*p*
Gender		0.529
Female	8 months (7,10)	
Male	10 months (6,12)	
Location		0.286
Head	9 months (7,10)	
Body	6 months (3,10)	
Tail	10 months (4,30)	
Overall survival calculated from primary diagnosis		< 0.0001
Synchronous	7 months (6,9)	
Metachronous	14 months (10,26)	
Overall survival calculated from diagnosis of metastases		0.235
Synchronous	7 months (6,9)	
Metachronous	4 months (3,8)	
Metastasis's locations^a^		0.032
Liver only	8 months (6,10)	
Lungs only	10 months (7,21)	
Both liver and lungs	10 months (7,22)	

^a^ Considering only the liver and lungs.

### 3.2 Linear/Parallel Ratio and Other Parameters of Metastases

**FIGURE 2:**
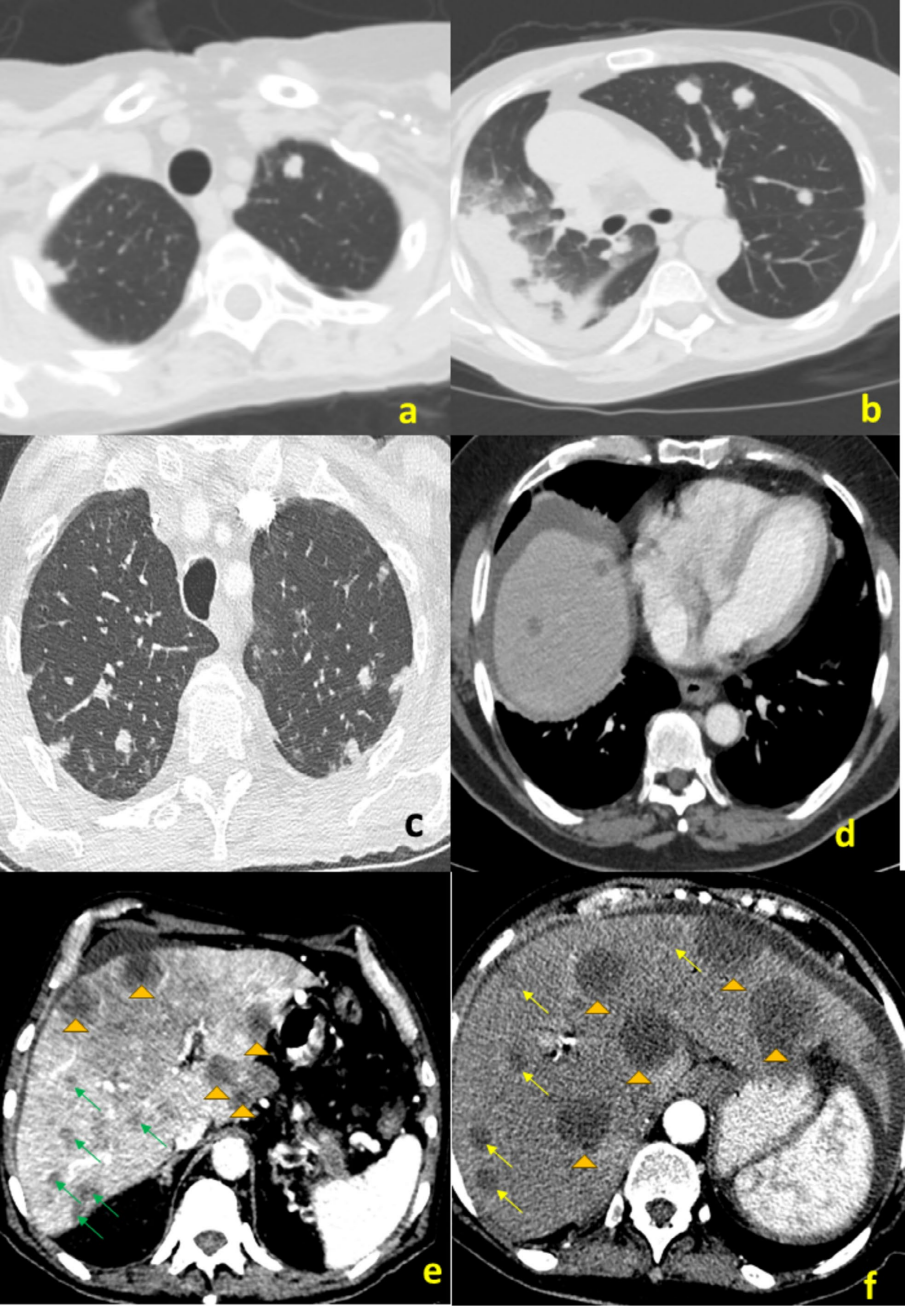
Computerized axial images of patients with pancreatic cancer metastases. A 70‐year‐old woman with a single wave of synchronous metastases (a and b). A 61‐year‐old man with a single wave of synchronous metastases (c). A 69‐year‐old man with a single wave of liver metastases (d). A 77‐year‐old man with two waves of synchronous liver metastases (e). A 68‐year‐old woman with two waves of metachronous metastases (f). The first wave is marked by arrowheads and the second by arrows.

A total of 1054 lung and 2898 liver metastases were identified (Table 3). In 80 patients (60.1%), liver‐only metastases were found; in 27 patients (20.3%), lung only; and in 26 patients (19.6%) both organs were involved. LPR was high in both organs, with a median of +1 (IQR 0.97,1) in the lungs and +0.98 (IQR 0.83,1) in the liver. Representative photos of this phenomenon are shown in Figure 2. The median number of lung metastases was 12 (IQR 5,36) and their median diameter was 6.5 mm (IQR 5.4,7.8). The median number of liver metastases was 11 (IQR 5,36.5) and their median diameter was 16 mm (IQR 12.4,21.9). Lung metastases were clustered to a median of 2 clusters (IQR 1,4) and liver metastases to 3 clusters (IQR 2,6). Comparisons of parameters of synchronous and metachronous metastases are shown in Table 4. Metachronous lung metastases were significantly fewer in number (median of 8 vs. 13, *p* = 0.009) and in number of clusters (median of 1 vs. 3, *p* = 0.015) compared to synchronous metastases. Such a difference was not found in the liver.

**TABLE 3:** Parameters of lung and liver metastases.

	Median lung (IQR 1,3)	Median liver (IQR 1,3)
Number	12 (5,36)	11 (5,36.5)
Liner/parallel ratio	1 (0.97,1)	0.98 (0.83,1)
Average metastasis diameter (mm)	6.5 (5.4,7.8)	16 (12.4,21.9)
Standard deviation (mm)	1.8 (0.5,2.8)	5.12 (3.5,7.7)
Largest metastasis (mm)	10 (7,13)	27 (20.5,37)
Number of clusters	2 (1,4)	3 (2,6)
Number of single metastases	0 (0,1)	1 (0,2)

**TABLE 4:** Comparisons of parameters of synchronous and metachronous metastases.

	Metachronous median (IQR 1,3)	Synchronous median (IQR 1,3)	*p*
Lung			
Total number per patient	8 (4.0,13.0)	13 (5.25,38.25)	0.009
Average size (mm)	6.2 (4.9,6.4)	6.6 (5.6,8.0)	0.069
STD (mm)	0.53 (0.15,0.69)	1.9 (0.9,2.9)	0.045
Largest metastasis (mm)	7.0 (5.5,7.2)	10.0 (7.0,13.75)	0.11
Number of clusters	1 (1.0,2.0)	3 (2.0,5.0)	0.015
Number of single metastases	0 (0.0,0.0)	0 (0.0,1.0)	0.21
Linear/parallel ratio	1 (0.93, 1.0)	0.9 (0.79, 1.0)	0.05
Liver			
Total number per patient	12.5 (6.0, 20.5)	10 (5.0,38.0)	0.498
Average size (mm)	17.5 (12.9,22.6)	15.1 (12.4,24.1)	0.622
STD (mm)	5.8 (3.6,6.9)	4.9 (3.5,7.9)	0.163
Largest metastasis (mm)	30 (24.8,35.0)	25 (20.0,39.5)	0.127
Number of clusters	3 (2–5.5)	3.5 (2.0,6.0)	0.418
Number of single metastases	1 (0,2)	1 (0.0,2.0)	0.747
Linear/parallel ratio	1 (1.0,1.0)	0.9 (0.78,1.0)	0.203

## 4 Discussion

In this study, we attempted to decipher the trajectory of PDAC lung and liver metastases. We used methodology that sorts metastases according to their diameters. This methodology allows rapid classification of metastasis routes as linear or parallel (or mixed) in a substantial number of patients. It is supported by genomic analysis and was used to decipher the clonal origin of synchronous and metachronous metastases in multiple tumor types [16, 17]. This concept may also be used to refine the definition of the oligometastatic state [18]. The first observation of the current study is that dissemination to both organs is almost exclusively via the linear route. This striking feature of PDAC is portrayed by the extremely high LPR of both liver and lung metastases (Table 3 and Figure 2). It means that almost all PDAC metastases arise directly from the primary, and that there is minimal, if any, maturation in the target organs. This observation is in accordance with molecular studies. Whole genome sequencing of 26 metastases from four patients showed identical driver gene mutations in the primary tumor and in its metastases [10]. In the largest series in this field, 95 metastases from 289 patients showed concordance in the mutation pattern of primary tumors and their metastases [11]. Accordingly, all metastases should be represented in the primary tumor, and most metastases can be classified by genetically identical waves. This linear spread is also supported by genomic analysis performed by Yachida et al. on rapid autopsies in seven individuals and showed that all metastases clonal population is represented within the primary tumor [19]. This high LPR was also found in malignancies of the thyroid and kidney [16]. Additionally, a median of two metastatic waves (IQR 1,4) was found in the lungs and three waves (IQR 2,6) in the liver. Only a few metastases (a median of 0, IQR 0,1 in the lungs and 1, IQR 0,2 in the liver) did not fit into any cluster. After landing in the lungs and liver, due to their genomic instability, metastatic cells continue to evolve independently. This process is not reflected in their diameters due to the poor longevity of patients at this state.

The high LPR of PDAC also means that metachronous metastases found after surgery with curative intent are already present at the time of surgery but are too small to be detected by current imaging modalities. This observation is supported by calculations of doubling time of liver metastases done by Amikura et al. in six patients after pancreatectomy [20]. These authors showed that at the time of pancreatectomy, liver metastases were present in all patients with “organ confined” disease and were as small as 0.14 mm.

In the same line is the second observation of this study. The OS rate of patients with metachronous metastases is better when calculated from the time of diagnosis (median of 14 months vs. 7 months, *p* < 0.0001). Nevertheless, when calculated from the time of metastases diagnosis, the difference disappears (median of 7 vs. 4 months, *p* = 0.235). This suggests that synchronous and metachronous metastases differ by a lead time of 7 months but not in clinical behavior (Table 2 and Figure 2). The extremely poor survival of patients with metachronous disease (median OS of 4 months) is well recognized in the literature. Median OS in this magnitude, especially in patients with liver recurrence, is well documented [8, 9, 21]. This phenomenon was termed “sudden rapid increase” by Collins et al., and it may be attributed to immunosuppression induced by major surgery and adjuvant therapies [22]. Since patients after treatment of a presumably localized disease are under careful surveillance, it is expected that their (metachronous) metastases would be diagnosed earlier compared to patients with synchronous disease (Table 4). Indeed, these patients had fewer lung metastases (median of 8 vs. 13, *p* = 0.009) and fewer waves of lung metastases (median of 1 vs. 3, *p* = 0.015). Such a difference was not found in the analysis of liver metastases, probably due to the superior sensitivity of CT in detecting metastases in the lungs.

It has been suggested that patients with lung metastases have better prognosis compared to patients with metastases at other sites [23, 24, 25]. Potential explanations for this phenomenon include less clinically significant location (compared to liver metastases that can potentially block the gastric outlet or the biliary system) and different molecular aberrations. In the current series patients with liver‐only metastases had significantly worse prognosis compared to patients with lung‐only metastases, but the survival curve of patients with both liver and lung metastases was almost identical to that of patients with lung‐only disease (Table 2 and supplementary material 3). The explanation of this phenomenon is not clear but could be related to metastases in other locations that were not analyzed in this study.

### 4.1 Limitations

This study is limited by the retrospective methodology and by the difficulty of manually measuring metastases diameters in the liver. The methodology used here also allows the clustering of later but faster growing metastasis with earlier but slower one. The accuracy of the LPR model is limited when the number of metastases is small and improves with an increasing number. No genomic analyses were performed in the current study, but its results are fully supported by genomic information in previous studies [10, 11].

### 4.2 Conclusions

This study showed that the metastatic spread of PDAC to both the liver and the lungs is almost exclusively via a linear trajectory, that is, directly from the primary tumor, and that patients with synchronous and metachronous metastases differ in lead time of metastases diagnosis but not in clinical behavior. Taken together, this means that liver or lung metastases are already present in most patients with PDAC at the time of initial diagnosis but are sometimes too small to be detected by current imaging modalities. The clinical implication of this is that local treatments given to patients with “no evidence of metastatic disease” may thus have a dramatic local effect but do not significantly modify the risk of developing later metastases. Any local treatment in PDAC must be accompanied by systemic treatment.

## Author Contributions

O.N.G. and S.N.G. developed the concept and wrote the manuscript. B.G. wrote the computer code and performed the statistics. A.P. and J.S. reviewed and edited the manuscript.

## Ethics Statement

The study was approved by the local IRB (approval number 0034‐23‐HMO). Patient's records were anonymized prior to analysis. No informed consent or additional ethical committee review was required.

## Conflicts of Interest

The authors declare no conflicts of interest.

## Supporting information


**Data S1** The complete dataset.

**Data S2** The computerized code used for calculation of the linear/parallel ratio (LPR).

**Data S3** Overall survival curves according to recurrence location.

## Data Availability

All the raw data is available in Supporting Information.
