# Supplementary figures and images for: The Clonal Trajectory of Liver and Lung Metastases in Pancreatic Ductal Adenocarcinoma
Source: Cancer Rep (Hoboken). 2025 May 9;8(5):e70228. doi: 10.1002/cnr2.70228 (PMC12063064; doi:10.1002/cnr2.70228)

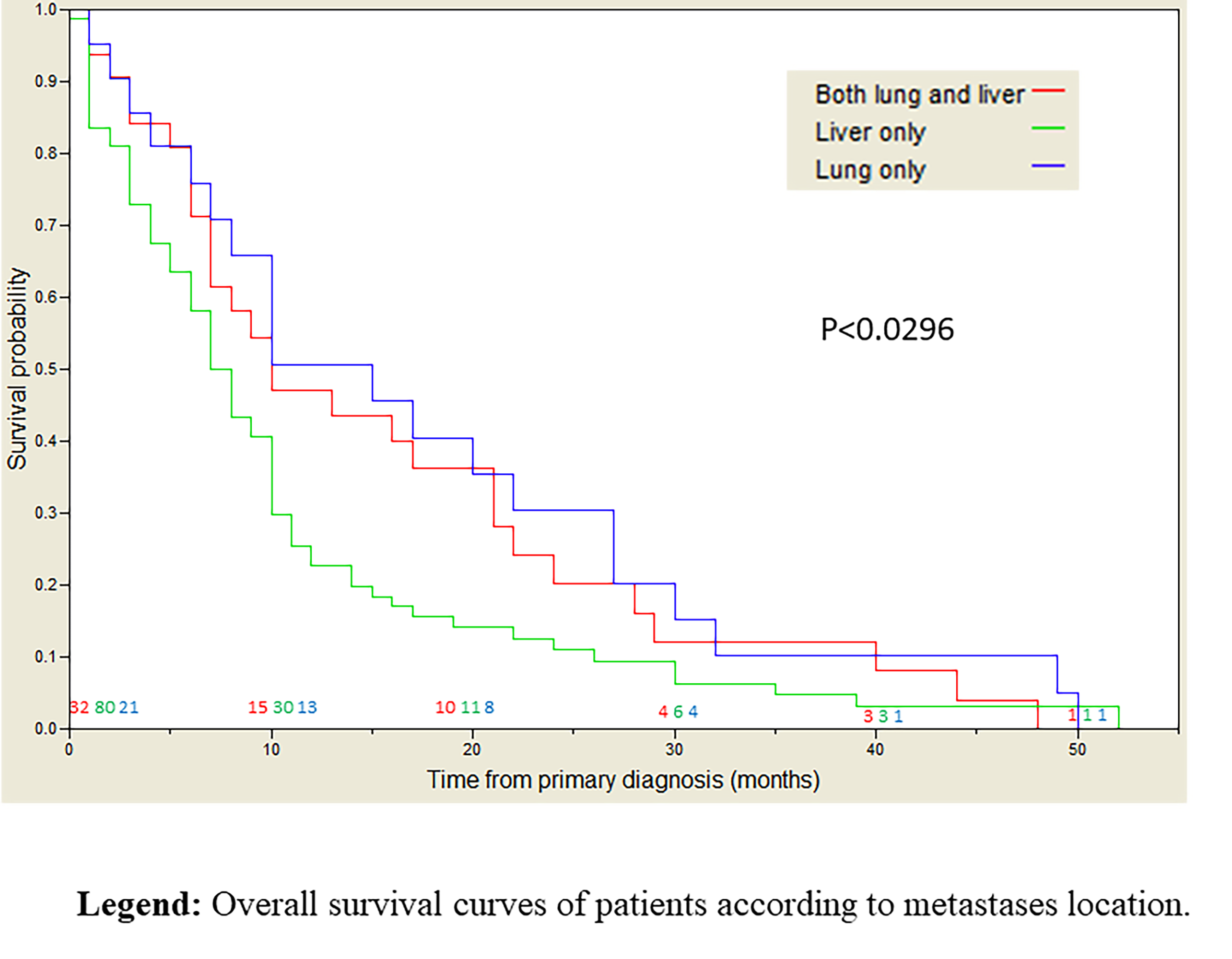

Supplement: Supplementary file 3 — Data S3 Overall survival curves according to recurrence location. [file CNR2-8-e70228-s001.tif]
